# Rapid adaptation of multisensory integration in vestibular pathways

**DOI:** 10.3389/fnsys.2015.00059

**Published:** 2015-04-16

**Authors:** Jerome Carriot, Mohsen Jamali, Kathleen E. Cullen

**Affiliations:** Department of Physiology, McGill UniversityMontreal, QC, Canada

**Keywords:** vestibule, vestibular nuclei, astronauts, internal model, vestibular diseases, sensorimotor, adaptation, sensory reweighting

## Abstract

Sensing gravity is vital for our perception of spatial orientation, the control of upright posture, and generation of our everyday activities. When an astronaut transitions to microgravity or returns to earth, the vestibular input arising from self-motion will not match the brain's expectation. Our recent neurophysiological studies have provided insight into how the nervous system rapidly reorganizes when vestibular input becomes unreliable by both (1) updating its internal model of the sensory consequences of motion and (2) up-weighting more reliable extra-vestibular information. These neural strategies, in turn, are linked to improvements in sensorimotor performance (e.g., gaze and postural stability, locomotion, orienting) and perception characterized by similar time courses. We suggest that furthering our understanding of the neural mechanisms that underlie sensorimotor adaptation will have important implications for optimizing training programs for astronauts before and after space exploration missions and for the design of goal-oriented rehabilitation for patients.

## Introduction

On earth, gravity provides a unique reference axis to which we can anchor our body orientation and monitor orientation changes. Indeed, as Lacquaniti et al. (2004) have noted, it is a force to which we are constantly exposed starting from the day we are born. The findings of theoretical as well as behavioral studies have led to the longstanding hypothesis that the brain builds an internal model of the expected sensory consequence of our own actions (Wolpert et al., 1998; Wolpert and Ghahramani, 2000; Herzfeld et al., 2014)—which is required for accurate spatial orientation, the control of posture, and the generation of precise movements. On earth, the expectation of the constant force of gravity is an inherent component of this internal model. By combining the information available from different modalities (i.e., the vestibular, proprioceptive and somatosensory as well as the visual systems) with its internal model, the brain can sense and anticipate the consequences of the force of gravity (reviewed in McIntyre et al., 1998; Zupan et al., 2002). During space exploration missions, however, gravity becomes minimal resulting in a mismatch between the brain's expectation of sensory consequence of movement and that actually experienced. This has important implications for astronauts. Specifically, astronauts show impaired balance, locomotion, gaze control, dynamic visual acuity, eye–head–hand coordination during the space flight (reviewed in Souvestre et al., 2008).

The effects of this decrease in gravity are most pronounced immediately after the transition to microgravity (Montgomery et al., 1993). When moving, astronauts not only experience impairments in sensorimotor performance but also report spatial disorientation and destabilizing sensations such as the feeling of have suddenly turned upside-down and/or difficulty in sensing the location of their own arms and legs (Souvestre et al., 2008). It is generally thought that these symptoms arise because the integration of sensory input from the vestibular system with that from the proprioceptive, somatosensory, and visual systems misinforms the brain relative to its existing (i.e., earth-based) “internal” model of the expected sensory consequences of motion (Freeman, 2000). This conflict between the brain's expectation of sensory feedback and the actual sensory feedback experience in microgravity is also thought to be the cause of space motion sickness experiences during the initial stages of space flight (Freeman, 2000; Oman and Cullen, 2014). Overall, nearly 70% of all astronauts experience impaired motor performance and/or space motion sickness (Lackner and Dizio, 2006). To develop new training and treatment approaches, it is important to understand the mechanisms that underlie these symptoms as well as those that are responsible for recovery.

As discussed below, our recent work provides 2 major advances in this area. First we have shown that neurons that sense “sensory conflict” from the otoliths can be found at the first stage of central vestibular processing (i.e., vestibular nuclei; Carriot et al., 2013, 2014, 2015), and that the cerebellum plays a key role in computing the difference between expected and actual vestibular input during active motion (Brooks and Cullen, 2013). Second, our work shows that when the vestibular input experienced during motion is altered relative to normal conditions, the dynamic re-weighting of multimodal inputs enables the sensorimotor adaptation observed during behavioral recovery.

## Neural correlates of sensorimotor adaptation: sensory conflict

**Figure 1:**
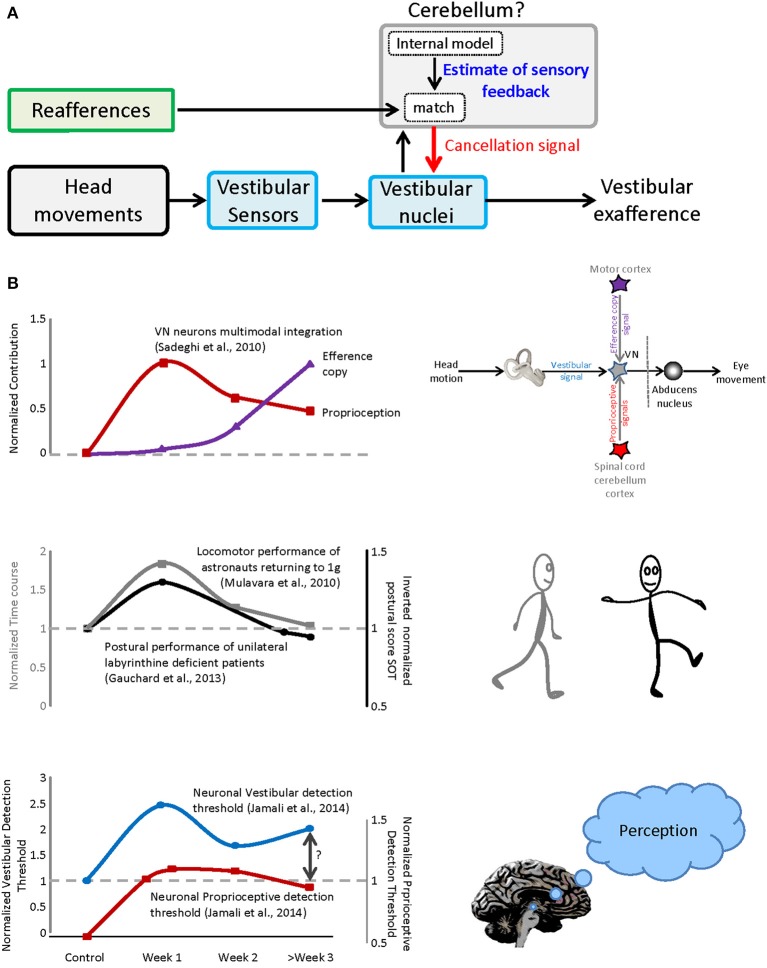
**Sensorimotor adaptation during vestibular compensation and re-entry. (A)** Proposed mechanism for the selective encoding of exafference. Vestibular reafference is canceled when the actual sensory input matches the expected sensory consequence of motor command. **(B)** Top panel: the time course of dynamic re-weighting of multimodal inputs during vestibular compensation. Middle panel: the time course of the behavioral adaptation for vestibular patients as well as astronauts after re-entry. Bottom panel: the contribution of vestibular and neck inputs to the neuronal detection thresholds over the course of recovery after vestibular loss.

In single unit recording studies we have shown that a subclass of neurons in the vestibular nuclei, which project to the spinal cord and to vestibular thalamus, respond preferentially to passive head movements. For example, during everyday activities, the otoliths are activated both by gravity and by our own self-motion (Carriot et al., 2014). In response to active motion, the otolith afferents in the 8th nerve send robust signals to the vestibular nuclei (Jamali et al., 2009). However, at the next stage of processing, this “reafferent” sensory input is canceled (Carriot et al., 2013, 2015). Our recent work further suggests that this cancelation is mediated by a mechanism that compares the expected consequences of self-generated movement (computed by an internal model located in the cerebellum) and the actual sensory feedback (Figure 1A). Notably, the un-canceled sensory input (“exafference”) resulting from passive movement is thought to allow the brain to compensate for unexpected postural disturbances and ensure perceptual stability (reviewed in Cullen, 2012). Such a mechanism is similarly consistent with the observation that impairments in balance, locomotion, gaze control, dynamic visual acuity, eye–head–hand coordination and perception are most serious during the initial phase of space flight and re-entry. Once the brain's internal model had been updated to account for the change in the forces gravity, it learns to expect a different pattern of input from the 8th nerve during motion that again ensures accurate motor control and perceptual stability.

## Neural correlates of sensorimotor adaptation: dynamic re-weighting of multimodal inputs

Indeed, although the symptoms of space motion sickness/spatial disorientation can initially be quite debilitating, they decrease over time (from 1 h to 4 days) such that astronauts are able to comfortably make voluntary head movements during a mission. Astronauts again experience these symptoms upon returning to Earth's 1-g environment. Because access to astronauts is more straightforward in this latter condition, it is more often the focus of quantitative studies. Interestingly, aspects of the motor performance observed after returning to 1-g environment astronauts are similar to those observed in the patients from acute unilateral vestibular peripheral loss (see also Mulavara et al., 2012). In both conditions, the actual vestibular feedback experienced during self-motion is initially different from that which is expected. Recent studies in our laboratory have provided insights into the neural mechanisms that underlie the adaptation of the sensori-motor integration following peripheral vestibular loss (Sadeghi et al., 2010, 2011, 2012; Jamali et al., 2014).

In particular, we discovered that compensation is mediated by the dynamic reweighting of inputs from different modalities (i.e., extravestibular versus vestibular) at the level of the single neurons that constitute the first central stage of vestibular processing. At least two types of extravestibular inputs can substitute for the lost vestibular input, (1) proprioception and (2) motor efference copy signals. As reviewed above, in normal conditions, vestibular responses to active motion are suppressed when there is a match between the brain's estimate of proprioceptive feedback and the actual sensory feedback. However, under normal conditions (1) passive stimulation of neck proprioceptors in isolation does not alter neuronal responses and (2) the generation of motor efference copy signals does not alter neuronal responses when the head is prevented from moving (i.e., in this condition there is a mismatch between expected and actual feedback). In contrast, following peripheral vestibular loss, neurons respond differently. First, robust response to passive stimulation of neck proprioceptors are rapidly unmasked in the early vestibular pathway (Figure 1B, top panel, red trace), can be linked to the compensation process as evidenced by faster and more substantial recovery of the resting discharge in proprioceptive-sensitive neurons (Sadeghi et al., 2010). Furthermore, when the head is restrained neuronal responses to motor efference copy are unmasked over the course of weeks (Figure 1B, top panel, purple trace).

## The dynamics of behavioral adaptation: vestibular compensation and re-entry

The time course of the dynamic re-weighting of multimodal information observed at the level of single neurons, follows a similar time course to the improvement observed in (1) patient performance following vestibular loss and (2) astronaut return back to earth (Figure 1B, compare top and middle panels). First, patients generally show significant improvement in postural performance in first days after lesion, with more gradual improvement seen within a 1–2 weeks (Figure 1B, middle panel, gray trace adapted from Gauchard et al., 2013). Early sensorimotor symptoms include significant head tilt in the roll plane toward the lesion and a tendency to deviate toward the lesioned side when walking (reviewed in Smith and Curthoys, 1989; Curthoys and Halmagyi, 1995). Second and similarly, astronauts show rapid sensorimotor learning in the first day after return, with more gradual improvement in the following weeks ultimately returning performance to pre-flight levels. Superimposed in Figure 1B (middle panel) for comparison is an example of the adaption that occurs following return from space flight (e.g., locomotor performance; Mulavara et al., 2010). A similar time course has been reported for balance control recovery (Paloski et al., 1992; Reschke et al., 1998), as well as perception, spatial orientation, eye–head and head-trunk coordination following re-entry (Glasauer et al., 1995; Bloomberg et al., 1997; Reschke et al., 1998; Bloomberg and Mulavara, 2003; Courtine and Pozzo, 2004; Clement and Wood, 2014).

## Neural correlates of perception: dynamic re-weighting of multimodal inputs

Recently, we have further demonstrated that, following partial vestibular loss, neurons at the first central stage of vestibular processing, in the vestibular nuclei, show increased variability in response to vestibular stimulation. This increase in variability does not improve over time and ultimately constrains neural detection thresholds (Jamali et al., 2014; Figure 1B, bottom panel, blue trace). As noted above, these neurons not only contribute to posture and balance via projections to the spinal cord, but also send information to the thalamus, and then on to regions of cerebral cortex. Accordingly, they likely make a vital contribution to the perception of spatial orientation and self-motion (reviewed in Cullen, 2012). This then raises the question: What mechanisms underlie the observed improvements in perceptual threshold? Indeed, we found that sensory substitution with extravestibular (i.e., proprioceptive; Figure 1B, bottom panel, red trace) inputs provides a neural substrate for improvements in self-motion perception following vestibular loss (e.g., Bergenius et al., 1996; Cutfield et al., 2011; Cousins et al., 2013) which similarly shows significant improvement over this same time frame.

## Conclusions

It is noteworthy that dynamic reweighting of extravestibular inputs occurs in the same neurons that sense “sensory conflict” during self-generated motion—namely at the first stage of central vestibular processing. Thus, sensorimotor adaptation in conditions where sensory input is altered (e.g., after vestibular loss of function or experiencing a new gravitational environment) appears to involve the simultaneous updating of internal models and the dynamic re-weighting of multimodal input. We speculate that the central nervous system utilizes a similar multimodal strategy to compensate for changes in gravity, experienced in both of these conditions. Our recent neurophysiological findings further provide a neural correlate for the benefits provided by rehabilitative strategies that take advantage of the convergence of multisensory cues during sensorimotor adaptation following vestibular loss. For example, standard rehabilitation techniques aimed at improving balance such as Cawthorne–Cooksey exercises incorporate the generation of head and body movements, which essentially provides multimodal stimulation (i.e., combined vestibular and proprioceptive; reviewed in Ricci et al., 2010).

### Conflict of interest statement

The authors declare that the research was conducted in the absence of any commercial or financial relationships that could be construed as a potential conflict of interest.

## References

[B1] Bergenius J. Tribukait A. Brantberg K. (1996). The subjective horizontal at different angles of roll-tilt in patients with unilateral vestibular impairment. Brain Res. Bull. 40, 385–390. discussion: 390–391. 10.1016/0361-9230(96)00131-1 8886363

[B2] Bloomberg J. J. Mulavara A. P. (2003). Changes in walking strategies after spaceflight. IEEE Eng. Med. Biol. Mag. 22, 58–62. 10.1109/MEMB.2003.1195697 12733460

[B3] Bloomberg J. J. Peters B. T. Smith S. L. Huebner W. P. Reschke M. F. (1997). Locomotor head-trunk coordination strategies following space flight. J. Vestib. Res. 7, 161–177. 10.1016/S0957-4271(96)00169-3 9178222

[B4] Brooks J. X. Cullen K. E. (2013). The primate cerebellum selectively encodes unexpected self-motion. Curr. Biol. 23, 947–955. 10.1016/j.cub.2013.04.029 23684973 PMC6100740

[B5] Carriot J. Brooks J. X. Cullen K. E. (2013). Multimodal integration of self-motion cues in the vestibular system: active versus passive translations. J. Neurosci. 33, 19555–19566. 10.1523/JNEUROSCI.3051-13.2013 24336720 PMC3858625

[B6] Carriot J. Jamali M. Brooks J. X. Cullen K. E. (2015). Integration of canal and otolith inputs by central vestibular neurons is subadditive for both active and passive self-motion: implication for perception. J. Neurosci. 35, 3555–3565. 10.1523/JNEUROSCI.3540-14.2015 25716854 PMC4339360

[B7] Carriot J. Jamali M. Chacron M. J. Cullen K. E. (2014). Statistics of the vestibular input experienced during natural self-motion: implications for neural processing. J. Neurosci. 34, 8347–8357. 10.1523/JNEUROSCI.0692-14.2014 24920638 PMC4051983

[B8] Clement G. Wood S. J. (2014). Rocking or rolling–perception of ambiguous motion after returning from space. PLoS ONE 9:e111107. 10.1371/journal.pone.0111107 25354042 PMC4213005

[B9] Courtine G. Pozzo T. (2004). Recovery of the locomotor function after prolonged microgravity exposure. I. Head-trunk movement and locomotor equilibrium during various tasks. Exp. Brain Res. 158, 86–99. 10.1007/s00221-004-1877-2 15164151

[B10] Cousins S. Kaski D. Cutfield N. Seemungal B. Golding J. F. Gresty M.. (2013). Vestibular perception following acute unilateral vestibular lesions. PLoS ONE 8:e61862. 10.1371/journal.pone.0061862 23671577 PMC3650015

[B11] Cullen K. E. (2012). The vestibular system: multimodal integration and encoding of self-motion for motor control. Trends Neurosci. 35, 185–196. 10.1016/j.tins.2011.12.001 22245372 PMC4000483

[B12] Curthoys I. S. Halmagyi G. M. (1995). Vestibular compensation: a review of the oculomotor, neural, and clinical consequences of unilateral vestibular loss. J. Vestib. Res. 5, 67–107. 10.1016/0957-4271(94)00026-X 7743004

[B13] Cutfield N. J. Cousins S. Seemungal B. M. Gresty M. A. Bronstein A. M. (2011). Vestibular perceptual thresholds to angular rotation in acute unilateral vestibular paresis and with galvanic stimulation. Ann. N.Y. Acad. Sci. 1233, 256–262. 10.1111/j.1749-6632.2011.06159.x 21951002

[B14] Freeman M. (2000). Challenges of Human Space Exploration. Chichester: Praxis Publishing Ltd.

[B15] Gauchard G. C. Parietti-Winkler C. Lion A. Simon C. Perrin P. P. (2013). Impact of pre-operative regular physical activity on balance control compensation after vestibular schwannoma surgery. Gait Post. 37, 82–87. 10.1016/j.gaitpost.2012.06.011 22824677

[B16] Glasauer S. Amorim M. A. Bloomberg J. J. Reschke M. F. Peters B. T. Smith S. L.. (1995). Spatial orientation during locomotion [correction of locomation] following space flight. Acta Astronaut. 36, 423–431. 10.1016/0094-5765(95)00127-1 11540973

[B17] Herzfeld D. J. Vaswani P. A. Marko M. K. Shadmehr R. (2014). A memory of errors in sensorimotor learning. Science 345, 1349–1353. 10.1126/science.1253138 25123484 PMC4506639

[B18] Jamali M. Mitchell D. E. Dale A. Carriot J. Sadeghi S. G. Cullen K. E. (2014). Neuronal detection thresholds during vestibular compensation: contributions of response variability and sensory substitution. J. Physiol. 592, 1565–1580. 10.1113/jphysiol.2013.267534 24366259 PMC3979612

[B19] Jamali M. Sadeghi S. G. Cullen K. E. (2009). Response of vestibular nerve afferents innervating utricle and saccule during passive and active translations. J. Neurophysiol. 101, 141–149. 10.1152/jn.91066.2008 18971293 PMC3815216

[B20] Lackner J. R. Dizio P. (2006). Space motion sickness. Exp. Brain Res. 175, 377–399. 10.1007/s00221-006-0697-y 17021896

[B38] Lacquaniti F. Bosco G. Gravano S. Indovina I. La Scaleia B. Maffei V.. (2004). Multisensory integration and internal models for sensing gravity effects in primates. Biomed. Res. Int. 2014:615854. 10.1155/2014/615854 25061610 PMC4100343

[B21] McIntyre J. Berthoz A. Lacquaniti F. (1998). Reference frames and internal models for visuo-manual coordination: what can we learn from microgravity experiments? Brain Res. Brain Res. Rev. 28, 143–154. 10.1016/S0165-0173(98)00034-4 9795191

[B22] Montgomery L. D. Parmet A. J. Booher C. R. (1993). Body volume changes during simulated microgravity: auditory changes, segmental fluid redistribution, and regional hemodynamics. Ann. Biomed. Eng. 21, 417–433. 10.1007/BF02368634 8214826

[B23] Mulavara A. P. Feiveson A. H. Fiedler J. Cohen H. Peters B. T. Miller C.. (2010). Locomotor function after long-duration space flight: effects and motor learning during recovery. Exp. Brain Res. 202, 649–659. 10.1007/s00221-010-2171-0 20135100

[B24] Mulavara A. P. Ruttley T. Cohen H. S. Peters B. T. Miller C. Brady R.. (2012). Vestibular-somatosensory convergence in head movement control during locomotion after long-duration space flight. J. Vestib. Res. 22, 153–166. 23000615 10.3233/VES-2011-0435

[B25] Oman C. M. Cullen K. E. (2014). Brainstem processing of vestibular sensory exafference: implications for motion sickness etiology. Exp. Brain Res. 232, 2483–2492. 10.1007/s00221-014-3973-2 24838552 PMC4130651

[B26] Paloski W. H. Reschke M. F. Black F. O. Doxey D. D. Harm D. L. (1992). Recovery of postural equilibrium control following spaceflight. Ann. N.Y. Acad. Sci. 656, 747–754. 10.1111/j.1749-6632.1992.tb25253.x 1599180

[B28] Reschke M. F. Bloomberg J. J. Harm D. L. Paloski W. H. Layne C. McDonald V. (1998). Posture, locomotion, spatial orientation, and motion sickness as a function of space flight. Brain Res. 28, 102–117. 10.1016/S0165-0173(98)00031-9 9795167

[B29] Ricci N. A. Aratani M. C. Dona F. Macedo C. Caovilla H. H. Gananca F. F. (2010). A systematic review about the effects of the vestibular rehabilitation in middle-age and older adults. Revista Brasileira de Fisioterapia 14, 361–371. 10.1590/S1413-35552010000500003 21180862

[B30] Sadeghi S. G. Minor L. B. Cullen K. E. (2010). Neural correlates of motor learning in the vestibulo-ocular reflex: dynamic regulation of multimodal integration in the macaque vestibular system. J. Neurosci. 30, 10158–10168. 10.1523/JNEUROSCI.1368-10.2010 20668199 PMC2933842

[B31] Sadeghi S. G. Minor L. B. Cullen K. E. (2011). Multimodal integration after unilateral labyrinthine lesion: single vestibular nuclei neuron responses and implications for postural compensation. J. Neurophysiol. 105, 661–673. 10.1152/jn.00788.2010 21148096 PMC3059170

[B32] Sadeghi S. G. Minor L. B. Cullen K. E. (2012). Neural correlates of sensory substitution in vestibular pathways following complete vestibular loss. J. Neurosci. 32, 14685–14695. 10.1523/JNEUROSCI.2493-12.2012 23077054 PMC3503523

[B33] Smith P. F. Curthoys I. S. (1989). Mechanisms of recovery following unilateral labyrinthectomy: a review. Brain Res. Brain Res. Rev. 14, 155–180. 10.1016/0165-0173(89)90013-1 2665890

[B34] Souvestre P. A. Landrock C. K. Blaber A. P. (2008). Reducing incapacitating symptoms during space flight: is postural deficiency syndrome an applicable model? Hippokratia 12(Suppl. 1), 41–48. 19048092 PMC2577399

[B35] Wolpert D. M. Ghahramani Z. (2000). Computational principles of movement neuroscience. Nat. Neurosci. 3(Suppl.), 1212–1217. 10.1038/81497 11127840

[B36] Wolpert D. M. Goodbody S. J. Husain M. (1998). Maintaining internal representations: the role of the human superior parietal lobe. Nat. Neurosci. 1, 529–533. 10.1038/2245 10196553

[B37] Zupan L. H. Merfeld D. M. Darlot C. (2002). Using sensory weighting to model the influence of canal, otolith and visual cues on spatial orientation and eye movements. Biol. Cybern. 86, 209–230. 10.1007/s00422-001-0290-1 12068787

